# Role of temporary arterial occlusion in subarachnoid hemorrhage outcomes: a prospective cohort study

**DOI:** 10.1590/acb387923

**Published:** 2023-12-04

**Authors:** Marcia Harumy Yoshikawa, Nícollas Nunes Rabelo, João Paulo Mota Telles, Leonardo Zumerkorn Pipek, Guilherme Bitencourt Barbosa, Natália Camargo Barbato, Antônio Carlos Samaia da Silva Coelho, Manoel Jacobsen Teixeira, Eberval Gadelha Figueiredo

**Affiliations:** 1 Universidade de São Paulo – São Paulo (SP) – Brazil.

**Keywords:** Intracranial Aneurysm, Subarachnoid Hemorrhage, Microsurgery, Intraoperative Complications, Treatment Outcome

## Abstract

**Purpose:**

Temporary arterial occlusion (TAO) is a widespread practice in the surgical treatment of intracranial aneurysms. This study aimed to investigate TAO’s role during ruptured aneurysm clipping as an independent prognostic factor on short- and long-term outcomes.

**Methods:**

This prospective cohort included 180 patients with ruptured intracranial aneurysms and an indication of microsurgical treatment. Patients who died in the first 12 hours after admission were excluded.

**Results:**

TAO was associated with intraoperative rupture (IOR) (odds ratio – OR = 10.54; 95% confidence interval – 95%CI 4.72–23.55; p < 0.001) and surgical complications (OR = 2.14; 95%CI 1.11–4.07; p = 0.01). The group with TAO and IOR had no significant difference in clinical (p = 0.06) and surgical (p = 0.94) complications compared to the group that had TAO, but no IOR. Among the 111 patients followed six months after treatment, IOR, number of occlusions, and total time of occlusion were not associated with Glasgow Outcome Scale (GOS) in the follow-up (respectively, p = 0.18, p = 0.30, and p = 0.73). Among patients who underwent TAO, IOR was also not associated with GOS in the follow-up (p = 0.29).

**Conclusions:**

TAO was associated with IOR and surgical complications, being the latter independent of IOR occurrence. In long-term analysis, neither TAO nor IOR were associated with poor clinical outcomes.

## Introduction

Ruptured intracranial aneurysms (IA) account for 80% of non-traumatic subarachnoid hemorrhage (SAH) cases^1^. SAH is a life-threatening condition, resulting in death in 30–40% of cases and, among most patients who survive, decreased quality of life and functional dependency^2^^,^ 3. Risk factors for intracranial aneurysm rupture include ethnicity (black race and Hispanic), hypertension, current smoking, alcohol abuse, use of sympathomimetic drugs, and aneurysm size > 7 mm^4^^,^ 5.

The treatment of ruptured IA may be endovascular or surgical. The surgical treatment indication relies on the patient’s age and neurologic status, aneurysm location, quality of the angiographic image, and need for vascularization with bypass^6^^–^ 9. Temporary arterial occlusion (TAO) is used during the surgical treatments of IA to facilitate aneurysm manipulation and clipping process, as well as to avoid and manage aneurysm intraoperative rupture (IOR)^7^^,^ 10 ^,^ 11.

Although being a widespread technique, TAO is associated with perioperative stroke in ruptured aneurysm cases, which is more likely to occur in patients with older age, Hunt-and-Hess grade IV and V, early surgery, and single prolonged clip placement^12^^,^ 13. Ischemic complications secondary to TAO also depend on the location of clip placement, occlusion duration, and the use of neuroprotective strategies, such as intraoperative neurophysiological monitoring of somatosensory evoked potentials^14^^–^ 16 and intraoperative hypothermia^17^^–^ 19.

Despite the relevance of TAO for IA surgery, most studies about the complications and the prognostic value of this technique are limited to the assessment of clinical and radiological evidence of perioperative stroke. Therefore, this study aimed to investigate the prognostic value of TAO in ruptured aneurysm surgery on short- and long-term outcomes.

## Methods

### Ethical standards

This prospective cohort study was conducted at the Central Institute of Hospital das Clínicas of Medical School at Universidade de São Paulo. The study was approved by the local ethics committee and conducted in accordance with relevant guidelines and regulations. All patients were fully informed about the study and provided informed consent, as approved by the local institutional review board.

### Patient selection

All patients admitted between January 2018 and May 2019 with subarachnoid hemorrhage were enrolled. The inclusion criteria were patients with the diagnosis of aneurysmal subarachnoid hemorrhage and indication of microsurgical treatment. Patients who died in the first 12 hours after admission, or had previous neurological disability were excluded.

### Clinical, surgical, and radiological evaluation

The clinical condition of patients with a ruptured aneurysm was assessed through Hunt-Hess Scale on admission. The clinical assessment also included comorbidities, history of smoking or alcohol abuse, previous SAH, and occurrence of surgical or clinical complications during hospitalization. Surgical data collected included: time from admission to surgery, presence of TAO, number of TAO in each case, total time of TAO, and occurrence of IOR. The radiological assessment included the modified Fisher (mFisher) on admission and the aneurysm location. The patients’ outcomes were prospectively evaluated at discharge and at six months after surgery. The patients were followed for six months and evaluated using the Glasgow Outcome Scale (GOS).

### Statistical analysis

Categorical data were described as frequencies, while continuous variables were described as mean (standard deviation) or median (interquartile distance), as appropriate under normal data analysis. To compare means of continuous variables, the T-Student or Mann-Whitney’s test was used, as appropriate. Dichotomous variables were analyzed using the χ^2^ test. A two-tailed alpha level of 5% was adopted. The analyses were performed using the GraphPad Prism 8.0.0 software (San Diego, United States of America).

## Results

### Demographic characteristics

The study included 180 patients. Sixty-nine (38.3%) patients lost follow-up and were excluded from the long-term analysis (n = 111). The short-term analysis included 131 females (72.7%) and 49 (27.2%) males, while the long-term analysis included 69 (62.1%) females and 42 (37.8%) males (Table 1).

**Table 1:** Baseline characteristics of the study population.

Short-term analysis		N (%)
	Total	180 (100%)
	Female	131 (72.7%)
	Male	49 (27.2%)
	Age median (range)	57.5 years old (21–88)
**Long-term analysis**		**N (%)**
	Total	111 (100%)
	Female	69 (62.1%)
	Male	42 (37.8%)
	Age median (range)	59 (21-86)
	Hypertension	60 (54%)
	DM	30 (27%)
	Smoking	41 (36.9%)
	Alcoholism	17 (15.3%)
	Previous SAH	12 (10.8%)

N: number of subjects; DM: diabetes mellitus; SAH: subarachnoid hemorrhage. Source: Elaborated by the authors.

The median age in the study population was 57.5 years old (range 21–88), while in the follow-up group it was 59 (range 21–86). Among the 111 patients followed, 60 (54%) had hypertension, 41 (36.9%) were smokers, 30 (27%) had diabetes mellitus, 17 (15.3%) had alcohol abuse history, and 12 (10.8%) had a previous SAH (Table 1).

### Clinical data

Time between ictus and surgery was assessed in 172 (95.5%) cases. Fifty-two patients (30.2%) were treated on day 0 to 2, 28 (16.2%) on days 3 and 4, 55 (31.9%) on day 5 to 10, and 37 (21.5%) on day 11 or later.

Hunt and Hess (HH) score was evaluated in all ruptured cases (n = 180). Among the ruptured cases that underwent TAO (n = 38), 20 (52.6%) had HH < 2, and 18 (47.3%) had HH > 2. Among the patients with ruptured aneurysms who did not undergo TAO (n = 142), 96 (67.6%) had HH < 2, and 46 (32.4%) had HH > 2 (Table 2).

**Table 2:** Analysis of clinical and radiological condition on admission and clinical outcomes in the follow-up of study’s participants.

Variables		TAO N (%)	No TAO N (%)
**Short-term analysis**			
	Total	38 (100)	142 (100)
HH on admission			
	HH ≤ 2	20 (52.6)	96 (67.6)
	HH > 2	18 (47.3)	46 (32.4)
mFisher on admission*			
	Grade 0–2	7 (18.4)	40 (28.1)
	Grade 3	10 (26.3)	40 (28.1)
	Grade 4	18 (55.2)	60 (42.2)
**Long-term analysis**			
	Total	21 (100)	90 (100)
GOS follow-up			
	GOS 1	6 (28.5)	31 (34.4)
	GOS 2	0 (0)	0 (0)
	GOS 3	0 (0)	7 (7.7)
	GOS 4	6 (28.5)	12 (13.3)
	GOS 5	9 (42.8)	40 (44.4)

TAO: temporary arterial occlusion; HH: Hunt and Hess; mFisher: modified Fisher classification; GOS: Glasgow Outcome Scale;

* mFisher was assessed in 140 out of 142 patients with ruptured aneurysm who underwent TAO. Source: Elaborated by the authors.

### Radiological data

The modified Fisher scale (mFisher) was assessed in 178 (98.8%) ruptured cases. All ruptured cases that underwent TAO (n = 38) had mFisher grade assessed: patients with grades 0–2 accounted for seven (18.4%), patients with grade 3 accounted for 10 (26.3%), and patients with grade 4 accounted for 18 (55.2%). Concerning the patients who did not undergo TAO (n = 142), mFisher was assessed in 140 (98.5%) cases, resulting in 40 (28.1%) patients with grade 0–2, 40 (28.1%) patients with grade 3, and 60 (42.2%) with grade 4 (Table 2).

The most common aneurysm location was the middle cerebral artery (MCA; n = 46), followed by the posterior communicating artery (PCoA; n = 41), anterior communicating artery (ACoA; n = 41), internal carotid artery (ICA; n = 19), and others (n = 30). Among the patients who underwent TAO (n = 38), the most common aneurysm location was also MCA (n = 17), followed by ACoA (n = 11), PCoA (n = 4), ICA (n = 2) and others (n = 4). Aneurysm location was not associated with clinical complications (p = 0.98), surgical complications (p = 0.67), or GOS in the follow-up (p = 0.14). Regarding patients who had TAO, aneurysm location was also not associated with either of these variables (p = 0.058, p = 0.74, and p = 0.95, respectively).

### Surgical data

TAO was performed in 38 (21.1%) out of the 180 cases included. TAO was performed in 22 (57.8%) females and 16 (42.1%) males. Regarding the type of craniotomy, the Pterional was the most frequent one, accounting for 20 cases, followed by Minipterional (n = 6), Frontopterional (n = 5), Nanopterional (n = 3), Frontal (n = 3), Pre-temporal (n = 1) and others (n = 1).

Twenty-eight (73.6%) patients had one temporary clip, seven had two (18.4%) temporary occlusions, and three (7.8%) had three or more. The total time of occlusion was assessed in 37 (97.3%) ruptured cases: 30 (78.9%) patients had TAO for ≤ 15 minutes, two (5.2%) for 15–20 minutes, and five (13.1%) for ≥ 20 minutes. Data on IOR was assessed in 37 (97.3%) cases and occurred in 15 (40.5%). The association between TAO and IOR was statistically significant (odds ratio – OR = 8.11; 95% confidence interval – 95%CI 4.72–23.55; p < 0.001).

### Clinical and surgical complications

Among the 180 patients included, 56 (31.1%) had one or more surgical complications, and 125 (69.4%) had one or more clinical complications. The most frequent clinical complications were vasospasm, hydrocephalus, intracranial hypertension, brain swelling, urine infection, pneumonia, hemiparesis, and death. The most frequent surgical complications were ischemia, cerebral bleeding (not related to IOR), and vascular injury (Table 3).

**Table 3:** Analysis of clinical and surgical complications of 180 patients.

Variables		TAO N (%)	No TAO N (%)
	Total	38 (100)	142 (100)
**Clinical complication**			
	Vasospasm	13 (34.2)	21 (14.7)
	Intracranial hypertension	10 (26.3)	9 (6.3)
	Hydrocephalus	4 (10.5)	27 (19)
	Brain swelling	8 (21)	17 (11.9)
	Urine infection	8 (21)	13 (9.1)
	Hemiparesis	9 (23.6)	22 (15.4)
	Death	13 (34.2)	32 (22.5)
	Other	20 (52.6)	57 (40.1)
**Surgical complication**			
	Ischemia	9 (23.6)	12 (8.4)
	Cerebral bleeding	3 (7.8)	4 (2.8)
	Vascular damage	2 (5.2)	1 (0.7)
	Other	8 (21)	12 (8.4)

N: number of patients; TAO: temporary arterial occlusion. Source: Elaborated by the authors.

In the group of patients with TAO (n = 38), clinical complications accounted for 31 (81.5%) cases, while surgical complications accounted for 18 (47.3%) cases. In the group without TAO (n = 142), clinical complications accounted for 94 (66.1%), while surgical complications accounted for 38 (26.7%).

TAO was associated with surgical complications (OR = 2.31; 95%CI 1.02–5.21; p = 0.02), but not with clinical complications (p = 0.1). The total time of occlusion was neither associated with surgical (p = 0.16) nor with clinical complications (p = 0.6), while the number of occlusions was associated with clinical complications (p = 0.04), but not with surgical complications (p = 0.09).

The group with TAO and IOR (n = 15) had 10 (66.6%) cases of clinical complications and seven (46.6%) cases of surgical complications. On the other hand, the group with TAO without IOR (n = 22) included 20 (90.9%) cases of clinical complication and 10 (45.4%) cases of surgical complication (Table 4). In the statistical analysis, the group with TAO and IOR did not differ from the group with TAO without IOR in terms of clinical (p = 0.06) and surgical (p = 0.94) complications (Table 4).

**Table 4:** Clinical and surgical complication in patients who underwent temporary arterial occlusion (TAO) stratified based on the occurrence of intraoperative rupture (IOR).

Variables	TAO with IOR N (%)	TAO without IOR N (%)	p-value
Total N	15 (100)	22 (100)	
Clinical complication	10 (66.6)	20 (90.9)	0.06
Surgical complication	7 (46.6)	10 (45.4)	0.94

Source: Elaborated by the authors.

### Follow-up

Among the 180 patients included in the short-term analysis, only 111 (61.6%) were followed six months later. Thirty-seven (33.3%) died after discharge (GOS 1), seven (6.3%) presented severe disability (GOS 3) and 49 (44.1%) had none or slightly significant disability (GOS 5). TAO was not associated with GOS in the follow-up (p = 0.84). IOR, number of occlusions, and total time of occlusion were also not associated with GOS in the follow-up (respectively p = 0.18, p = 0.30, and p = 0.73).

The HH score was associated with the GOS score in the follow-up (95%CI 0.54–0.91; p = 0.01). Each 1 point in HH was associated with OR = 0.7 for a worse outcome in the follow-up (95%CI 0.54–0.91; p = 0.01). The regression analysis demonstrated that IOR (in patients with TAO) is not associated with GOS in the follow-up (p = 0.29).

## Discussion

In recent years, endovascular coiling has gained increased space as IA treatment. However microsurgical clipping remains an important and widely used method for this condition. One of the main uses of TAO is for the prevention and management of IOR. IOR is a common intraoperative complication, accounting for 3.2–50% of cases of aneurysms treated surgically and associated with poor outcomes^20^^–^ 24. The use of TAO during IA’s surgery is currently widely accepted by the neurosurgical community, but evidence of its role as a prognostic factor on short- and long-term outcomes (beyond perioperative evidence of stroke) is still lacking.

There is evidence that TAO may induce cerebral ischemia, which extension would mainly depend on the duration of occlusion, location of clip placement, and presence of collateral vessels^12^^,^ 24 ^–^ 26. Most studies agree that the risk of neurological impairment is increased when the TAO takes 20 minutes or longer^11^^,^ 12 ^,^ 24 ^–^ 26. Complications associated with TAO may not only be secondary to occlusion duration, but also to the intravascular mobilization of atherosclerotic plaques and inadvertent lesions of perforation vessels.

In the current study, TAO had a significant association with IOR and surgical complications. Since TAO is usually required to manage IOR, patients who underwent TAO were expected to demonstrate intra- and post-operative complications secondary to IOR, and not to TAO per se. However, there was no significant difference in clinical or surgical complications between the groups that underwent TAO and had IOR and the groups that had TAO and no IOR.

Regarding the total duration of TAO, it was neither associated with short-term outcomes (clinical and surgical complications) nor long-term outcomes. Griessenauer et al.^25^ investigated the impact of TAO on long-term outcomes of patients with SAH and did not find any association between the total time of occlusion and the GOS on follow-up. Kim et al.^27^ did not find any association between total TAO duration and clinical outcomes at discharge in patients with ruptured anterior choroidal artery (AchoA) aneurysms treated surgically either. Similar to our cohort, Griessenauer et al.^25^ and Kim et al.^27^ studied populations who underwent TAO for ? 20 minutes, which is considered a safe period. The lack of association between the total time of occlusion and outcomes endorses the recommendation of using TAO for ? 20 minutes. Other studies with SAH patients presented the same lack of association^12^^,^ 28.

In this study, except for hydrocephalus cases, the group that had TAO presented higher rates of all clinical complications when compared to the group that did not have it. Previous investigations have suggested that the additional manipulation required for the temporary clip placement might be associated with vasospasm in patients with multiple and ruptured aneurysms^29^^,^ 30. Some experimental studies have also achieved the same findings, indicating the damage to the endothelium and tunica media induced by temporary clips as potential causes^31^^,^ 32. However, evidence on the role of TAO on vasospasm pathogenesis remains scarce and further investigations are needed.

### Limitations

This study has some limitations. First, most patients had temporary clips for less than 15 minutes, which is considered a safe time frame according to most authors. As a result, the absence of association between TAO duration and clinical outcomes may be related to the use of TAO for brief periods. Second, the loss of follow-up resulted in a 38.3% decrease in our initial sample size and, consequently, a decrease in the statistical power of our analysis. Finally, in tandem with other studies in the area, GOS was used to measure clinical outcomes in the follow-up; however, it is a limited tool to assess subtle changes in brain function.

## Conclusion

TAO was associated with surgical complications in patients with ruptured aneurysms, regardless of IOR occurrence. In the follow-up, neither TAO nor IOR was associated with GOS. Increased total time of occlusion and number of occlusions were not associated with poor outcomes after six months.

## Data Availability

The data will be available upon request.
